# Intensify the application of ZnO-based nanodevices in humid environment: O_2_/H_2_ plasma suppressed the spontaneous reaction of amorphous ZnO nanowires

**DOI:** 10.1186/1556-276X-9-281

**Published:** 2014-06-02

**Authors:** Chun-Yen Lai, Tzu-Chiao Chien, Ting-Yi Lin, Teng Ke, Shih-Han Hsu, Yun-Ju Lee, Chien-ying Su, Jeng-Tzong Sheu, Ping-Hung Yeh

**Affiliations:** 1 Department of Physics, Tamkang University, Tansui 25137, Taiwan; 2 Department of Materials Science and Engineering, National Chiao Tung University, Hsinchu 30050, Taiwan

**Keywords:** Spontaneous reaction, Humidity reaction, a-ZnO, Plasma passivation

## Abstract

In this work, we have demonstrated that amorphous ZnO nanobranches (a-ZnO NBs) could spontaneously react from the crystalline ZnO NWs (c-ZnO NWs) at specific humid environment. The spontaneous reaction mechanism and result can be analyzed by humidity controlling and optical microscope (OM)/scanning electron microscope (SEM)/Kelvin probe force microscopy (KPFM)/transmission electron microscopy (TEM) system. We can make the c-ZnO NWs spontaneous reaction happen at different humid environments and suppress the a-ZnO NBs spontaneous reaction by oxygen/hydrogen plasma surface passivation. The hydrogen plasma surface treatment also can improve the UV sensing sensitivity more than twofold. This work provides the mechanism and methods of the a-ZnO NBs spontaneous growth and offers the passivation treatment for strengthening and enhancing ZnO-based nanodevice application in humid environment and UV light detection, respectively.

## 

As one of the most important materials, ZnO has been extensively applied in numerous purposes which include optics, energy [1,2], piezo-phototronics [3-6], Schottky contact nanosensors [7-9], biomedical sciences [10,11], and spintronics [12]. Due to diverse and abundant nanostructures and a great potential in nanotechnology, a great number of novel ZnO nanodevices such as piezoelectric power generators [13-16], field-effect transistors (FET) [17,18], ultraviolet photodetectors [19], Schottky diodes [6,20-22], switches [21], and flexible piezotronic strain sensors [23] are gradually under research. Those devices, moreover, are expected to operate in various environments; therefore, maintaining their great performance and stability for an extended period of time is required. Due to this reason, nanostructures of ZnO in different atmospheres have become an interesting topic to study. According to several research articles, amorphous ZnCO_3_ thin films and nanowires could be formed due to the defacing of ZnO nanostructures by moisture and the small amount of CO_2_ in the atmosphere [24,25]. In this work, we would figure out the mechanisms of the spontaneous reaction and prove the efficacy of c-ZnO NWs surface passivation that would suppress the spontaneous reaction.

## Methods

Crystalline ZnO NWs were prepared with the common procedures presented in a previous research article [26]. Briefly, a proper amount of ZnO powders, treated as the precursor and loaded on an alumina boat, were placed at the center of an alumina tube which was set in a furnace to serve as the reaction chamber. A furnace was heated to 1,475°C and held at that temperature for 4.5 h and the gas, Argon, flowed through an alumina tube at a flow rate of 50 sccm to carry ZnO vapors to the end of an alumina tube for NWs growing. Then, the tube was cooled down to room temperature under a continuous argon flow. Crystalline-ZnO NWs were placed on the substrates (cleaned by standard processes) by homemade nanomanipulator. After that, the different samples were loaded into the various humidity conditions waiting for periodically observation. The samples were analyzed and measured by Zeiss SIGMA FESEM (Oberkochen, Germany)/Veeco Dimension 3100 SPM/JEM-2100 F FETEM (Plainview, NY, USA), and Agilent B1500A (Santa Clara, CA, USA).

## Results and discussion

**Figure 1:**
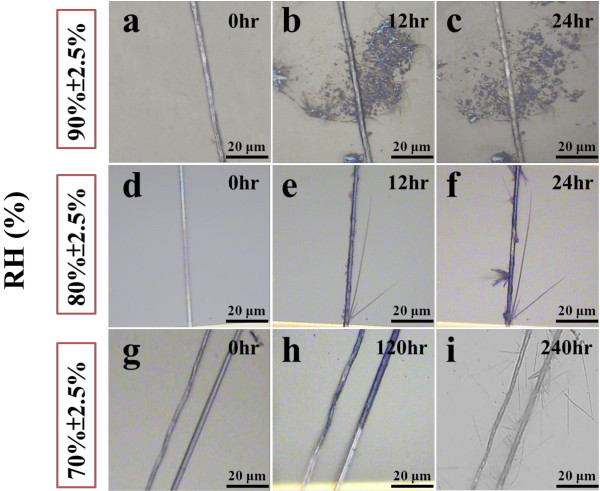
**The spontaneous reaction of ZnO nanobranches (NBs) can be observed by optical microscope (OM).** The morphology of ZnO NBs is varied with time and humidity (70% ± 2.5%, 80% ± 2.5%, and 90% ± 2.5%). **(a, b, c)** In high humidity (90% ± 2.5%), plenty of ZnO particles can be found around the ZnO NWs about 12 h. **(d, e, f)** When the humidity is around 80% ± 2.5%, a few ZnO NBs can be found within 12 h. **(g, h, i)** In low humidity (70% ± 2.5%), there are no ZnO NBs can be formed until 240 h.

The spontaneous reaction of a-ZnO nanobranches (NBs) could be observed by optical microscopy (OM); the morphology of a-ZnO NBs was varied with time and humidity (70% ± 2.5%, 80% ± 2.5%, and 90% ± 2.5%), as shown in Figure 1, which implied that the reliable performance of ZnO nanodevices might be deteriorated or even broken down by absorbing abundant H_2_O molecules. In high humidity (90% ± 2.5%), there are some ZnO particles that could be seen around the ZnO NWs, as illustrated in Figure 1a,b,c. In low humidity (70% ± 2.5%), a great number of thin and needle-like a-ZnO NBs formed from the c-ZnO NWs; the length and direction of the a-ZnO NBs were varied and random as shown in Figure 1g,h,i. Furthermore, when the value of humidity is around 80%, some flawed spots would become nucleate points; most a-ZnO NBs were grown from those nucleate points. Compare these three conditions; the a-ZnO NBs could be grown much faster and thicker in humidity 80% ± 2.5% (within 12 h) than in humidity 70% ± 2.5% (almost 10 days). So the percentage of humidity will be an important parameter for the morphology of spontaneous reaction.

**Figure 2:**
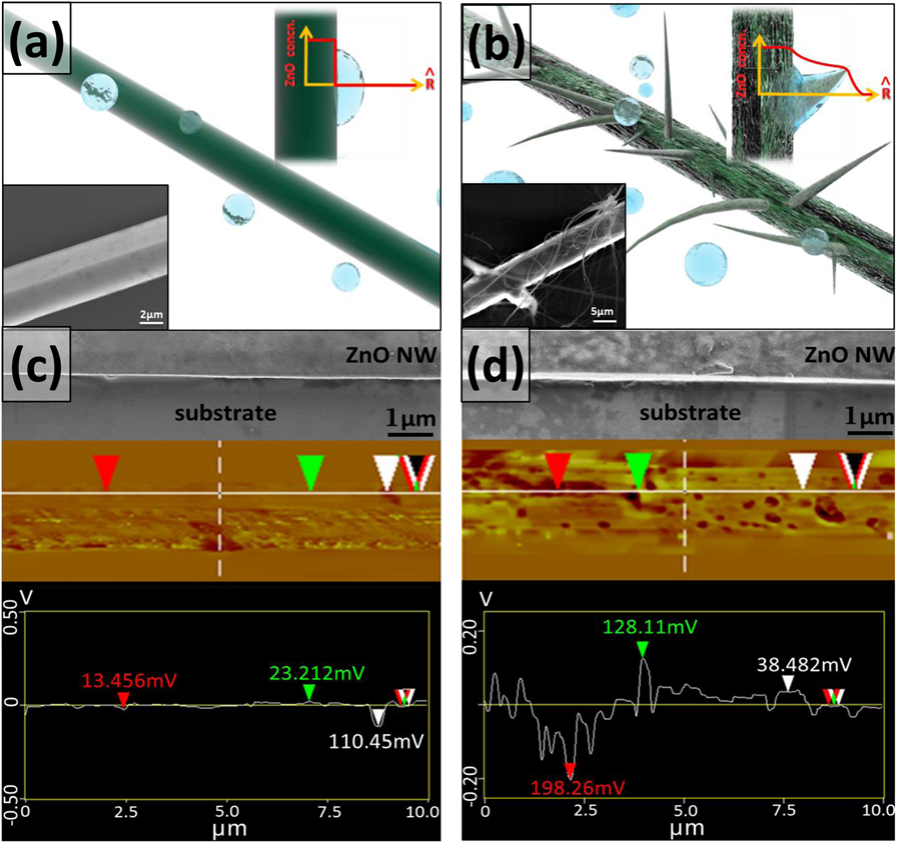
**The spontaneous reaction mechanism of a-ZnO NBs is illustrated. (a)** A uniform c-ZnO NWs (dark green rod) placed in the moisture environment surrounded by H_2_O molecules (light blue bubbles). The c-ZnO NW has uniform ZnO concentration which can be seen from the inset (ZnO concentration versus radius). **(b)** After H_2_O molecules absorbed at the surface of c-ZnO NWs, the Zn^2+^ ions would be dissolved from the surface of c-ZnO NWs and became aqueous solution diffused away from the c-ZnO NWs. When the Zn^2+^ ions and the ZnO NBs start to segregate out from the moisture solution and cause to grow from the main body of the original ZnO NWs, respectively (inset). **(c, d)** The surface potential was measured before and after moisture treatment.

The reaction mechanism of a-ZnO NBs can be studied by scanning electron microscopy (SEM) analysis as illustrated in Figure 2a,b. The H_2_O molecules (light blue bubbles) would be absorbed at the surface of c-ZnO NWs (the dark green rod) because the c-ZnO NWs are placed in the humid environment, as shown in the inset of Figure 2a. When the concentration of the H_2_O molecules was reaching certain level, the moisture would dissolve the specific spots of the c-ZnO NWs surface. Due to the interaction between the surface of c-ZnO NWs and moisture solution, the radial concentration of Zn^2+^ ion would be changed because Zn^2+^ ions gradually dissolve and diffuse from the original c-ZnO NWs surface into the moisture solution. When the concentration of Zn^2+^ ion in moisture solution meets the saturation condition, the Zn^2+^ ions start to segregate out from the moisture solution; the a-ZnO NBs cause to grow from the main body of the original c-ZnO NWs, which can be seen in Figure 2b. If the dimension of the original c-ZnO NWs is sufficient, the dissolving and diffusing effects can be maintained for a long period; the a-ZnO NBs will keep growing and forming ultra-long a-ZnO NBs. Normally, a-ZnO NBs would be spontaneously grown from specific size of c-ZnO NWs, such as around hundreds of nanometers. In high humidity, however, it is difficult for a-ZnO NBs to segregate from the moisture solution, which means that the Zn^2+^ ion concentration in moisture solution is not high enough to meet the condition of saturation forming a-ZnO NBs. That is why the ultra-long a-ZnO NBs cannot be seen in high humidity (90% ± 2.5%).

(1) $$\mathrm{Zn}O_{\left(s\right)}+H_{2}O_{\left(l\right)}↔\mathrm{Zn}{\left(\mathrm{OH}\right)}_{2\left(s\right)}$$

(2) $$\mathrm{Zn}{\left(\mathrm{OH}\right)}_{2\left(s\right)}+H_{2}O_{\left(l\right)}↔\mathrm{Zn}{\left(\mathrm{OH}\right)}_{3\left(\mathrm{aq}\right)}^{-}+H^{+}$$

(3) $$\mathrm{Zn}{\left(\mathrm{OH}\right)}_{2\left(s\right)}+2H_{2}O_{\left(l\right)}↔\mathrm{Zn}{\left(\mathrm{OH}\right)}_{4\left(\mathrm{aq}\right)}^{2-}+2H^{+}$$

**Figure 3:**
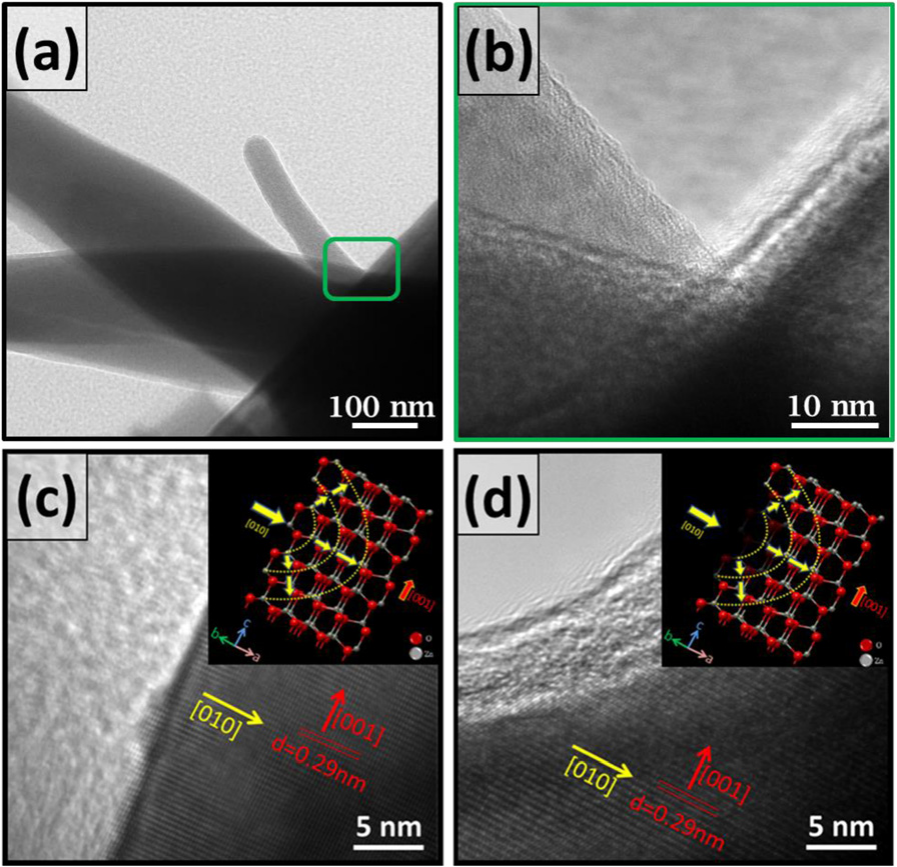
**The spontaneous growth of a-ZnO NBs. (a)** The a-ZnO NBs became new growth areas; amorphous nanostructures are around the a-ZnO NBs. **(b)** There are also amorphous layers on the c-ZnO NW near the roots of a-ZnO NBs. **(c)** ZnO NWs exhibit a single crystalline feature with the growth along [001] direction. **(d)** There are surface caves can be found on the c-ZnO NW due to the humidity influence; the dissolution direction is along [010].

The main reactions can be understood by the previous equations [27-29]; there are several reactive intermediates like Zn(OH)_4_^2−^, Zn(OH)_2_, or Zn(OH)_3_^−^, which depend on the specific parameters such as the concentration of Zn^2+^ ion, the amount of H_2_O molecules, and the pH value. Further investigation, the spontaneous growth mechanism of a-ZnO NBs can be studied through the c-ZnO NWs surface potential measurement by using Kelvin probe force microscope (KPFM) tapping mode. The surface potential of c-ZnO NWs can be changed due to the humidity absorption. Before humidity treatment, the surface morphology and potential were smooth and almost constant (around 10 to 25 mV variation) by SEM and KPFM analysis, respectively (Figure 2c). After humidity treatment, the surface morphology and potential were rough and varied (around 198.26 mV variation), respectively (Figure 2d). This surface potential variation might induce the a-ZnO NBs spontaneous growth.The spontaneous growth of a-ZnO NBs can be studied by the transmission electron microscopy (TEM) system, as illustrated in Figure 3. The a-ZnO NBs can be confirmed as an amorphous structure; the a-ZnO NBs will become new growth areas to keep extending the length of the a-ZnO NBs or growing extra a-ZnO NBs, as illustrated in Figure 3a, and there are amorphous layers around the c-ZnO NW near the roots of a-ZnO NBs, as shown in Figure 3b. The c-ZnO NW exhibit good crystalline feature with the growth along [001] direction, as shown in Figure 3c. The surface caves can be found on the c-ZnO NWs surface, and those caves might be the humidity influence; the dissolution direction is along [010], as shown in Figure 3d.

**Figure 4:**
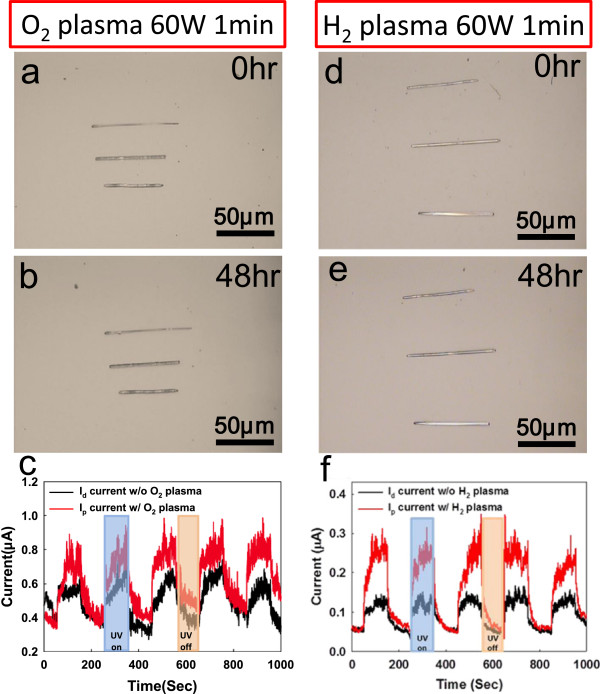
**The c-ZnO NWs have been passivated by O**_**2**_**/H**_**2 **_**plasma treatment. (a, b)** c-ZnO NW with O_2_ plasma (60 mW, 1 min) passivation has maintained the original forms after 48 h humidity (80% ± 2.5%) treatment. **(c, d)** ZnO NWs with H_2_ plasma (60 mW, 1 min) passivation also have no a-ZnO NBs spontaneous reaction from the ZnO NWs. **(e)** For O_2_ plasma treatment, the UV sensing ability can be improved. **(f)** For H_2_ plasma treatment, the UV sensing ability of ZnO nanodevice also enhanced more than two fold.

For general condition, the spontaneous reaction is loath to reveal in the ZnO NWs application; therefore, we have suppressed the spontaneous reaction from our c-ZnO NWs devices by using surface oxygen/hydrogen plasma treatment [30]. Due to dangling bonds on the surface of c-ZnO NWs, H_2_O molecules would be absorbed on the c-ZnO NWs surface much easier. If we can prevent the H_2_O molecule from the surface of the c-ZnO NWs, the spontaneous reaction might not happen and the ZnO nanodevices would maintain the functionality and performance. The c-ZnO NWs surface passivation can slow down the interaction between the moisture solution and c-ZnO NWs surface; the passive c-ZnO NWs would not have the spontaneous reaction in the same humidity treatment, as seen in Figure 4a,b,c,d). Using oxygen/hydrogen plasma (60 mW) to occupy the oxygen vacancy, the a-ZnO NBs spontaneous reaction can be suppressed, compared with the unpassive c-ZnO NWs. Both O_2_ and H_2_ plasma can improve the UV detection ability, but the H_2_ plasma treatment has stronger enhancement, compared with O_2_ plasma treatment, as shown in Figure 4e,f. The UV sensing ability of ZnO NWs device also can be enhanced more than twofold by H_2_ plasma treatment, as shown in Figure 4f. The plasma treatment not only can suppress the spontaneous reaction but also can enhance the UV sensing ability of the ZnO NWs devices.

## Conclusions

We have demonstrated that c-ZnO NWs would spontaneously react in humid environment; a-ZnO NBs structure can be grown with the appropriate humidity (around 70% to 85%). The spontaneous reaction is due to the interaction between the H_2_O molecules and the surface of c-ZnO NWs. The spontaneous reaction mechanism also can be proved by OM, SEM, KPFM, and TEM analyses. Finally, the a-ZnO NBs spontaneous reaction also can be suppressed by oxygen/hydrogen plasma surface passivation treatment; the plasma treatment could passivate the surface of the c-ZnO NWs from the H_2_O molecule. The spontaneous reaction would not happen, and the ZnO NWs devices would maintain the functionality; for UV sensing, the sensitivity could be enhanced more than twofold by using H_2_ plasma treatment. This research not only provides the mechanism and methods of the a-ZnO NBs spontaneous reaction but also offers the passivation treatment for intensifying ZnO NWs device application in humid environment and enhancing the UV light detection sensitivity.

## Competing interests

The authors declare that they have no competing interests.

## Authors’ contributions

The work presented here was performed in collaboration of all authors. CYL and TCC figured out the mechanism about this research. TYL and TK did the O_2_/ H_2_ plasma treatment on the c-ZnO NWs. CYL, SHH and YJL did the FESEM and HRTEM analysis. CYS and JTS did the KPAFM analysis. PHY organized the article. All authors read and approved the final manuscript.
